# Taiwan Tangerine Extracts Attenuate Amyloid Pathology and Neurotoxicity in Alzheimer’s Disease Cellular Model

**DOI:** 10.1002/alz70861_107912

**Published:** 2025-12-23

**Authors:** Amadou Wurry Jallow, Ta‐Wei Liu, Ching‐Kuo Lee, Yung‐Feng Lin

**Affiliations:** ^1^ Taipei Medical University, Taipei City Taiwan; ^2^ Taipei Medical university, Taipei City Taiwan; ^3^ Taipei Medical University, Taipei City, CA USA

## Abstract

**Background:**

Alzheimer’s disease (AD) is marked by excessive amyloid‐beta (Aβ) accumulation, leading to neurotoxicity and cognitive decline. The APP Swedish/Indiana (APP Swe/Ind) mutation enhances Aβ production, making it a relevant cellular model for AD pathology. *Citrus depressa* Hayata, or Taiwan tangerine, is traditionally used in Taiwanese medicine as a remedy for respiratory ailments. However, its effects on APP mutant‐expressing neurons remain unclear. This study investigates the anti‐amyloidogenic and neuroprotective effects of *Citrus depressa* extracts (CDE) in APP Swe/Ind‐transfected SH‐SY5Y cells and explores potential therapeutic mechanisms.

**Method:**

Human SH‐SY5Y neuroblastoma cells were transfected with APP Swe/Ind to induce Aβ overproduction. The effects of CDE on Aβ levels were evaluated using Western blot and ELISA, while cell viability was assessed by CCK‐8 assay. Bioactive targets of CDE were predicted using SEA, TargetNet, and SwissTargetPrediction. Functional roles were explored through Gene Ontology (GO) and KEGG analyses. The STRING database was used for protein‐protein interaction (PPI) network analysis. Gene expression data from the GSE5281 dataset, including 87 AD patients and 74 controls, were analyzed to validate target relevance.

**Result:**

Ethyl oleate, a key compound in CDE, significantly reduced both intracellular and extracellular Aβ levels in APP Swe/Ind cells and improved cell viability. It also preserved synaptic protein expression, indicating protection against Aβ‐induced synaptic dysfunction. Nine overlapping targets were identified across the prediction databases. GO and KEGG analysis linked these targets to pathways in neuroprotection and lipid metabolism. Expression analysis revealed that genes such as CNR1, HMGCR, FAAH, and PRKCD were significantly dysregulated in AD patients. Hierarchical clustering and heatmap analysis showed distinct expression profiles between AD and control groups. PPI network analysis identified HMGCR, CNR1, MGLL, FAAH, EPHX2 and ALOX5 as key therapeutic targets.

**Conclusion:**

Ethyl oleate from *Citrus depressa* extract reduces Aβ accumulation and protects synaptic integrity in an AD cellular model. These findings support the potential of *Citrus depressa* as a natural therapeutic candidate for AD treatment.

